# Sex differences in orbitofrontal connectivity in male and female veterans with TBI

**DOI:** 10.1007/s11682-015-9379-3

**Published:** 2015-04-12

**Authors:** Erin McGlade, Jadwiga Rogowska, Deborah Yurgelun-Todd

**Affiliations:** 1 Department of Psychiatry, University of Utah School of Medicine, Salt Lake City, UT USA; 2 VISN 19 MIRECC, Salt Lake City, UT USA; 3 University of Utah Brain Institute, Salt Lake City, UT USA

**Keywords:** Traumatic Brain Injury, Orbitofrontal, Functional connectivity, Aggression

## Abstract

More female soldiers are now serving in combat theaters than at any other time. However, little is known about possible sex differences underlying the neuropathology and manifestation of one of modern war’s signature injuries, traumatic brain injury (TBI). The paucity of information regarding sex differences in TBI is particularly evident when examining changes in executive function and emotion regulation associated with post concussive events. The current study objective was to observe whether patterns of orbitofrontal (OFC) functional connectivity would differ between female veterans with TBI and their male counterparts. The study further sought to determine whether OFC connectivity might be differentially associated with clinical measures of aggression and hostility. Seventeen female veterans and 24 male veterans, age 18 to 25, who met criteria for TBI completed resting state magnetic resonance imaging (MRI) and clinical assessment measures. Imaging data were analyzed using left and right seed regions of the OFC, and regression analyses were conducted to observe the relationship between resting state connectivity and self-reported aggression. Females and males in this study differed in OFC connectivity, with females demonstrating greater connectivity between left and right OFC and parietal and occipital regions and males demonstrating greater connectivity between left and right OFC and frontal and temporal regions. Significant associations between resting state connectivity and clinical measures were found only in male veterans. These findings suggest that TBI may interact with sex-specific patterns of brain connectivity in male and female veterans and exert divergent effects on clinical profiles of aggression post-injury.

## Background

Since 1950, more than eight million United States service members have been deployed to areas of conflict, including Korea, Southeast Asia, the Gulf, Iraq, and Afghanistan (Department of Veterans Affairs 2013; Congressional Budget Office 2012). Moreover, there are currently over two million female veterans and 20 million male veterans living in the United States (United States Department of Veterans Affairs 2014a), many of whom have been deployed, while others have served stateside. A significant number of veterans experience a host of physical and psychiatric complications, including traumatic brain injury (TBI), posttraumatic stress disorder (PTSD) and depression (MDD). In 2013, the Department of Veterans Affairs (VA) Healthcare served nearly 6 million veterans with a cost of over $40 billion (United States Department of Veterans Affairs 2014b). The integration of more female veterans into combat theaters recently has drawn attention to potential sex differences in the incidence and recovery from TBI, as well as a host of other comorbid disorders. Improved knowledge regarding sex differences related to TBI and comorbid psychiatric disorders is especially important now, as more female veterans are likely to access VA services. Understanding the role of sex-related effects has the potential to inform the assessment and treatment needs of female veterans, which may demonstrate both similarities and differences from male veterans with TBI (Luxton et al. 2010; Olff et al. 2007).

TBI has been associated with diffuse and focal brain changes to structures and networks underlying cognitive-emotional integration. Recent neuroimaging modalities have provided important techniques for characterizing neurobiological changes associated with TBI and its related behavioral symptoms (Matthews et al. 2011; Shenton et al. 2012; McDonald et al. 2012; Ross 2011). A significant portion of neuroimaging studies with TBI have focused on diffusion tensor imaging (DTI), a technique that has shown increased sensitivity in detecting axonal injury common in TBI (Arfanakis et al. 2002; Bazarian et al. 2007). For example, a recent study by Fakhran and colleagues used medical records to identify patients between the ages of 10 and 50 who had an identified mild TBI (mTBI) and completed DTI scans (47 male and 22 female) (Fakhran et al. 2014). Fakhran and colleagues used criteria including loss of consciousness for less than 1 min and posttraumatic amnesia for less than 30 min. The majority of participants sustained mTBI through sports injuries or motor vehicle accidents. Analyses showed that males with mTBI differed on fractional anisotropy (FA) values only in the uncinate fasciculus, suggesting changes in the fiber tract that connects limbic regions to the orbitofrontal cortex (OFC). A number of studies also have used functional MRI (fMRI) approaches to assess changes in brain connectivity using resting-state activity (Palacios et al. 2013; Sours et al. 2013). However, thus far no resting-state functional connectivity studies have been performed to examine sex differences in veterans with TBI.

While a number of investigations have reported sex differences in clinical response associated with TBI, the data are limited and often contradictory (Colantonio et al. 2010; Covassin et al. 2012). For example, Hoge and colleagues (2008) completed an epidemiological study of combat-related TBI and found that individuals who reported mTBI were more likely to be male, a finding consistent with earlier work from Bruns and Hauser (2003). However, an examination of post-concussive symptomatology (PCS) in a sample of 275 veterans with in-theater mTBI found that PCS reports were unrelated to sex, similar to a study of civilian TBI that showed no effect for gender on post-injury sequelae (Renner et al. 2012). The question remains, therefore, whether a new study cohort would demonstrate sex-related differences in post-concussive behaviors and to what extent these differences may be mediated by unique brain-based functional connectivity.

One important potentially sex-linked consequence associated with TBI is an increase in aggressive behavior or feelings. Brain injury has been linked to a range of behavioral sequelae, including aggression (Wood and Thomas 2013; Malkesman et al. 2013; Arciniegas and Wortzel 2014; Gallaway et al. 2012). Estimates suggest that up to 33.7 % of individuals with TBI exhibit aggressive behavior during the first 6 months post-injury (Tateno et al. 2003), although estimates are difficult due to variable definitions of aggression (Kim et al. 2007). Research also suggests that overall males tend to be more physically aggressive than females throughout the lifespan from childhood (Cleverley et al. 2012) to adulthood (Archer 2009). Gallaway and colleagues studied active duty Army soldiers and found that male soldiers reported significantly more minor and severe physical aggression (e.g., throwing something at someone, pushing, shoving, hitting, or, less frequently, using a knife/gun on someone) compared to female soldiers (Gallaway et al. 2012), whereas an investigation by Afari and colleagues examined self-reported aggression and found that male and female veterans report similar levels of aggressive behavior (Afari et al. 2015). Another challenge to understanding aggressive behavior in veterans and service members is that research in this area includes individuals with behavioral diagnoses such as intimate partner violence (Tharp et al. 2014; LaMotte et al. 2014; Love et al. 2014; Bradley 2007), irritable depression, and PTSD (Campbell et al. 2015; Flanagan et al. 2014; Reardon et al. 2014; Karairmak and Guloglu 2014; Angkaw et al. 2013; Elbogen et al. 2013). Indeed, the co-occurrence of TBI and PTSD has raised considerable debate in recent years with a number of investigators arguing that the two diagnoses frequently co-occur, however blurred the directionality of the etiology may be (Brenner et al. 2010; Carlson et al. 2011; King 2008; Morissette et al. 2011; Shandera-Ochsner et al. 2013).

It is evident that comorbid conditions including PTSD have raised significant challenges for the identification and interpretation of brain changes that may be associated with TBI in veterans. However, these behavioral presentations are likely closely related to the integrity of functional brain circuitry and critical to functional outcomes of the TBI patient. To address the question of co-morbidity associated with sex-related TBI profiles we have adopted a behavioral domain approach that has recently been recommended by the NIMH (Cuthbert 2014). This strategy focuses on clinical dimensions of human behavior rather than on categories of psychiatric disorders. (Craske 2012; Goodkind et al. 2015). Within this conceptual framework psychiatric disorders are seen as varied points or dimensions on the continua of human behavior rather than discrete groupings (Cuthbert 2014). The methodology provides a means for examining how neural circuits may be associated with different behavioral functions (Cuthbert and Insel 2013). In line with this new approach, the current study focuses on the dimension of aggression and its relationship to OFC connectivity.

As noted, the study of aggression has been of particular importance in veteran samples given high rates of TBI and the comorbidity of TBI and aggression (Rao et al. 2009; Wood and Thomas 2013). Moreover, it has been suggested that aggression can occur when an injury such as a TBI results in OFC damage, which affects the OFC’s ability to inhibit aggressive or other emotional responses generated by the amygdala and the rest of the limbic system (Wood and Thomas 2013; Rudebeck et al. 2008; Rolls 2004). Given its location in the brain, the OFC is also a structure highly susceptible to TBI. While its vulnerability to penetrating TBI has been well documented (Damasio 1994), more recent evidence suggests that coup contrecoup forces, such as those frequently observed in motor vehicle accidents, also place the OFC at significant risk of contusion as it scrapes along the bony ridge inferior to the prefrontal cortex (Taber et al. 2006; McDonald et al. 2002). A number of investigations have shown that the OFC has been strongly related to aggression as well as emotion dysregulation (Antonucci et al. 2006; Beyer et al. 2014; Mehta and Beer 2010; Grafman et al. 1996; Miczek et al. 2007; Gansler et al. 2011), particularly in instances where there are deficits in the top-down abilities of the OFC to modulate aggressive impulses or urges (Siever 2008). This subregion of the prefrontal cortex is also implicated in an array of executive processes ranging from valuation judgments to the decoding of environmental cues. Furthermore, damage to the OFC has been shown to result in psychosocial and executive deficits (Varney and Menefee 1993) as well as impairment in social and emotional cognition (Cicerone and Tanenbaum 1997) and reinforcement learning (Rolls 2004). The current investigation therefore focused on the connectivity of the OFC as it relates to measures of aggression in veterans with TBI.

The increasing number of female veterans in the United States and prior research showing associations between TBI, aggression, and the OFC clearly show sex differences in TBI and aggression as an area in need of further research. Given the key role the OFC plays in executive function and emotion regulation including aggression and its implication in the pathophysiology of TBI, the current study examined OFC functional connectivity in female and male veterans with TBI to determine if differences in connectivity patterns could be seen based on sex. Further, due to the high comorbidity of psychiatric disorders in TBI, regression analyses were completed to examine the association between aggression and OFC connectivity by sex. Based on prior research, we hypothesized that connectivity from the OFC will be different in female veterans compared to male veterans with TBI. Furthermore, we hypothesized that male and female veterans with TBI would differ in associations between measures of aggression and regions of interest using the OFC as the seed region.

## Methods

### Participants

All procedures followed were in accordance with the ethical standards of the responsible committee on human experimentation (institutional and national) and with the Helsinki Declaration of 1975, and the applicable revisions at the time of the investigation. Informed consent was obtained from all participants for being included in the study.

Study participants were recruited from the George E. Wahlen Health Care System and the greater Salt Lake City area. The Institutional Review Boards at the University of Utah and the George E. Wahlen Department of Veterans Affairs (VA) Medical Center approved this study. Participants in the study consisted of 17 female veterans and 24 male veterans aged 18–55 who met criteria for TBI. Individuals were excluded from the study if they had any major sensorimotor handicaps (e.g., deafness, blindness, paralysis); an estimated full scale IQ < 80; history of claustrophobia, autism, schizophrenia, anorexia nervosa or bulimia; history of electroconvulsive therapy; or metal fragments or implants that would be contraindicated in an MRI. Table 1 displays female and male veteran participant demographic information. Participants completed clinical and mood symptom measures in addition to neuroimaging.

**Table 1:** Demographic features of female and male veterans with TBI

	Females with TBI *n* (%)	Males with TBI *n* (%)
	*N* = 17	*N* = 24
Age	40.0 (SD = 11.15)	37.75 (SD = 9.59)
Race		
White	14 (82)	21 (88)
African American	1 (6)	0 (0)
Hispanic	2 (12)	3 (13)
Education (in years; mean, SD)	15.06 (SD = 2.51)	14.33 (SD = 2.10)
Education by Grade		
12	3 (18)	4 (17)
13	2 (12)	3 (13)
14	4 (24)	12 (50)
15	1 (6)	0 (0)
16	3 (18)	2 (8)
18	2 (12)	1 (4)
19	1 (6)	1 (4)
20	1 (6)	1 (4)
Marital status (married/not married) ***	3/14	15/9
Marital status by category ***		
Married	3 (18)	15 (62)
Remarried	0 (0)	0 (0)
Widowed	0 (0)	0 (0)
Separated	0 (0)	0 (0)
Divorced	9 (53)	3 (13)
Never been married	5 (29)	6 (25)
Missing	0 (0)	0 (0)
Children		
Yes	12 (71)	17 (71)
No	5 (29)	7 (29)
Rank		
E-1 to E-4	11 (65)	13 (57)
E-5 and above	5 (29)	10 (43)
Missing	1 (6)	1 (0)
Diagnosis		
Major Depressive Disorder (MDD)	2 (12)	1 (4)
Posttraumatic Stress Disorder (PTSD)	6 (35)	4 (17)
Comorbid MDD and PTSD	6 (35)	11 (46)

*p ≤ 0.07; **p ≤ 0.05; ***p ≤ 0.01

### Assessment measures

In addition to the Structured Clinical Interview for DSM-IV Patient Version (SCID-P), veterans completed the Ohio State University TBI Identification Method (OSU-TBI-ID), Hamilton Depression Rating Scale (HAM-D), Hamilton Anxiety Scale (HAM-A), Profile of Mood States (POMS), Buss-Perry Aggression Questionnaire (BPAQ), and Displaced Aggression Questionnaire (DAQ). (See Table 2.)

**Table 2:** Clinical and mood measure for female and male veterans with TBI

	Female with TBI *n* = 17		Male with TBI *n* = 24		
	Mean	SD	Mean	SD	*P*
HAM-A	12.00	7.04	12.08	9.61	0.98
HAM-D	9.41	5.46	10.50	8.15	0.64
POMS total	42.29	26.50	40.29	35.73	0.85
POMS tension	14.12	8.48	11.42	5.84	0.23
POMS depression	14.18	11.75	14.21	12.10	0.99
POMS anger	10.77	7.41	9.29	7.18	0.53
POMS vigor	16.41	6.57	14.75	5.46	0.38
POMS fatigue	14.41	4.78	11.13	7.47	0.12
POMS confusion	11.06	5.07	8.92	4.94	0.18
Buss perry physical aggression**	16.47	6.97	21.10	6.14	0.04
DAQ revenge planning	16.06	4.67	22.25	14.05	0.09
DAQ behaviorally displaced aggression	19.12	10.95	24.30	13.19	0.21

*p ≤ 0.07; **p ≤ 0.05; ***p ≤ 0.01

The OSU TBI-ID was administered to quantify presence, number, and severity of lifetime TBI. Veterans were considered to have a TBI if they reported an injury event to the head followed by an alteration or loss of consciousness; severity of TBI was classified based on Belanger and colleagues (Belanger et al. 2009). Specifically, mTBI was defined as an injury event with an alteration of consciousness (AOC) up to 24 hours or a loss of consciousness (LOC) of 0 to 30 minutes. Moderate TBI was defined as an injury event with AOC between 24 hours and 7 days or LOC between 30 minutes and 24 hours. Severe TBI was defined as AOC greater than 7 days or LOC greater than 24 hours. (See Table 3.) Participants completed a structured diagnostic interview (SCID-P) with a psychologist or board certified psychiatrist to establish MDD/PTSD diagnoses. Additionally, diagnoses were confirmed via consensus meetings and inter-rater reliability was consistently high (kappa > 0.90).

**Table 3:** Incidence of TBI in female veterans with TBI compared to male veterans with TBI

	Female with TBI *n* =17	Males with TBI *n* = 24
Mild TBI		
Frequency (positive for mTBI)	17 (100 %)	24 (100 %)
Moderate TBI		
Frequency (positive for moderate TBI)	1 (6 %)	1 (4 %)
Severe TBI		
Frequency (positive for severe TBI)	0 (0 %)	1 (4 %)
Time since most recent TBI mean months	96.35	154.50
Time since most severe TBI Mean months	147.18	204.00

Veterans completed the HAM-D and HAM-A to assess depressive and anxious symptomatology at the time of scanning. The HAM-D has shown utility in determining the level of depression before, during, and after treatment (Hamilton 1960). It is based on the clinician’s interview with the patient and probes symptoms such as depressed mood, guilty feelings, suicide, sleep disturbances, anxiety, and weight loss. Research has demonstrated a validity coefficient of .85 (Reynolds and Mazza 1998). The HAM-A is a rating scale developed to quantify the severity of anxiety symptomatology, and it is often used in psychotropic drug evaluations (Hamilton 1969).

Participants also completed the BPAQ and DAQ to assess self-reported aggression. The BPAQ is a 29-item self-report instrument that measures four aspects of aggression (physical, verbal, anger, and hostility) with established validity and reliability (Corcoran and Fisher 2000). A test retest reliability score of 0.80 has been reported along with a Cronbach alpha score of .89 (Buss and Perry 1992). The DAQ is a 31-item self-report questionnaire that measures three factors of aggression: angry rumination, revenge planning, and a behavioral dimension that estimates an overall tendency toward displaced aggression (Denson et al. 2006). Denson and colleagues reported a test retest reliability score of .77 (0.75 to 0.80 for individual scales) and a Cronbach alpha score of .95 (Denson et al. 2006).

### Magnetic resonance imaging

All imaging was performed at the Utah Center for Advanced Imaging Research (UCAIR) at the University of Utah using a 3 T Siemens Trio scanner. To control for gross structural brain changes all participants completed a structural MR scan, which was read by a neuroradiologist. Structural imaging data were acquired using a T1-weighted 3D MPRAGE GRAPPA sequence acquired sagittally using a 12-channel head coil with TE/TR/TI = 3.38 ms/2.0 s/1.1 s, 8° flip, 256 × 256 acquisition matrix, 256 mm2 FOV, 160 slices, and 1.0 mm slice thickness.

An 8-min resting-state scan, which included a total of 240 image volumes, was performed for each participant. BOLD echo planar images (TR = 2.0 s, TE = 28 ms, GRAPPA parallel acquisition with acceleration factor = 2, 40 slices at 3 mm slice thickness, 64 × 64 matrix) were obtained during a resting-state condition, and participants were asked to remain awake and let thoughts drift without maintaining focus. The stimulus computer was synchronized to the onset of the first BOLD image via fiber optic pulse emitted by the scanner.

### Image analysis

Functional MRI images were analyzed using SPM8 (Wellcome Department of Imaging Neuroscience, University College, London, UK) and Data Processing Assistant for Resting-State fMRI (DPARSF) software running in Matlab (MathWorks, Natick, MA, USA). The first 10 volumes of functional images were discarded for the signal equilibrium and to allow for the participants’ adaptation to scanning noise. Initially, the data underwent rigid body realignment to correct for head movement using rigid body motion correction procedure (Friston et al. 1996). We calculated 6 motion parameters – three for displacement (in x, y, and z) and three for rotation (pitch, roll, or yaw). The exclusion criteria were 2° or 2 mm in either the rotational or translational plane. No participants were excluded on this basis. There was no statistically significant difference in average motion being observed between the study groups. The realigned images were then normalized to an EPI template in Montreal Neurological Institute (MNI) stereotactic space. Normalized images were re-sampled into 3 mm cubic voxels and then spatially smoothed using an isotropic Gaussian kernel with 4 mm full width at half maximum (FWHM). Next, linear regression was used to reduce the effect of nuisance signals (motion parameters, global signal, and signals derived from cerebrospinal fluid and white matter) (Fox et al. 2005; Weissenbacher et al. 2009) and temporal band-pass filtering (0.01–0.08 Hz) was applied to reduce the effect of both very low and high frequency physiological noise.

After pre-processing steps, the seed regions for the left and right OFC were derived from the Automated Anatomical Labeling (AAL) atlas (Tzourio-Mazoyer et al. 2002) and the functional maps were computed by using a standard seed-based whole brain correlation method. For each seed region, the time series of the voxels within the seed region were averaged to generate the reference time series. Functional connectivity maps were computed by using a standard seed-based whole brain correlation method. For each seed region, the time series of the voxels within the seed region were averaged to generate the reference time series. For each subject and each seed region, the correlation coefficient was computed between the reference time series and the time course of each voxel of the brain. Correlation coefficients were converted to z-values using Fisher’s r-to-z transform (Mayer et al. 2011). The individual z-values were entered into a one-sample *t*-test in SPM8 to determine brain regions showing significant functional connectivity to the left and right OFC within each group (*p* < .05, FDR corrected; cluster size k > 20 voxels). Factorial analyses similar to those described in Whalley and colleagues (Whalley et al. 2009) were performed for the two groups controlling for both age and gender for the left and right OFC separately.

Finally, in order to determine the relationship between acute anxiety and depression on resting-state functional connectivity measures, regression analyses were completed between BOLD signal data and the scores on HAM A, HAM D, Buss Perry Physical Aggression, Displaced Aggression Questionnaire Behaviorally Displaced Aggression, and Displaced Aggression Questionnaire Revenge Planning (*p* < .005, and k > 20 voxels).

## Results

### Clinical and demographic findings in female and male veterans with TBI

Between group analyses were completed comparing 17 female veterans with TBI to 24 male veterans with TBI, as assessed using the OSU TBI-ID and established definitions of TBI (Belanger et al. 2009). All male and female veterans in the study met criteria for a history of mTBI; 1 male and 1 female reported a history of moderate TBI; and 1 male veteran reported a history of severe TBI. Analyses revealed no statistically significant between group differences in incidence of TBI, time since most recent TBI, or time since most severe TBI (Table 3). Veterans showed no significant between group difference in age (females mean age = 40.0, SD = 11.15; male mean age = 37.75, SD = 9.59) or education (female mean years of education = 15.06, SD = 2.51; male mean years of education = 14.33, SD = 2.10). (See Table 1.) Female and male veterans with TBI did not differ on race, children, or rank. Male and female veterans with TBI did differ significantly on current marital status (p = 0.006). More males with TBI reported being married at time of assessment, whereas more females with TBI reported being divorced.

Of the females with TBI, 2 met diagnostic criteria for MDD without PTSD, 6 met diagnostic criteria for PTSD without MDD, and 6 met diagnostic criteria for comorbid MDD and PTSD. Of the males with TBI, 1 met diagnostic criteria for MDD without PTSD, 4 met diagnostic criteria for PTSD without MDD, and 11 met diagnostic criteria for comorbid MDD and PTSD. (See Table 1.) Chi-square analyses showed no significant between-group differences on rates of MDD, PTSD, or comorbid MDD and PTSD (all *p* > 0.05).

On clinical measures, male veterans reported significantly more physical aggression compared to female veterans (*p* = 0.04). Male veterans also trended toward increased revenge planning compared to female veterans (*p* = 0.09). However, male and female veterans with TBI did not evidence significant differences in anxiety, depression, POMS scores, or behaviorally displaced aggression. (See Table 3.)

### Connectivity differences between female and male veterans with TBI

**Fig. 1:**
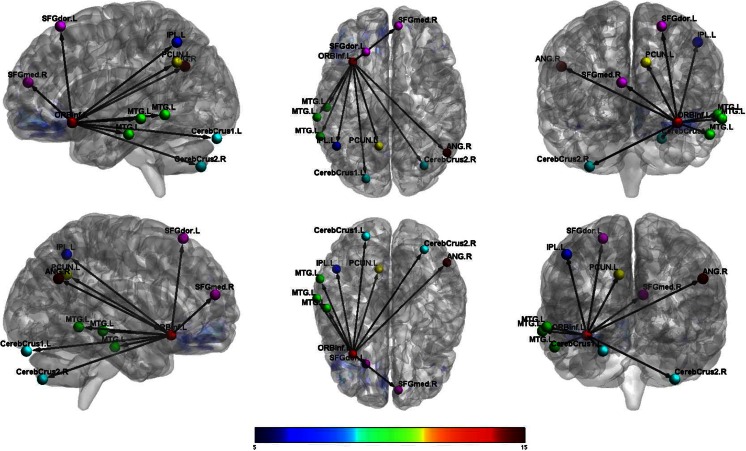
Female veterans with TBI: functional connectivity between left orbitofrontal cortex and other regions

**Fig. 2:**
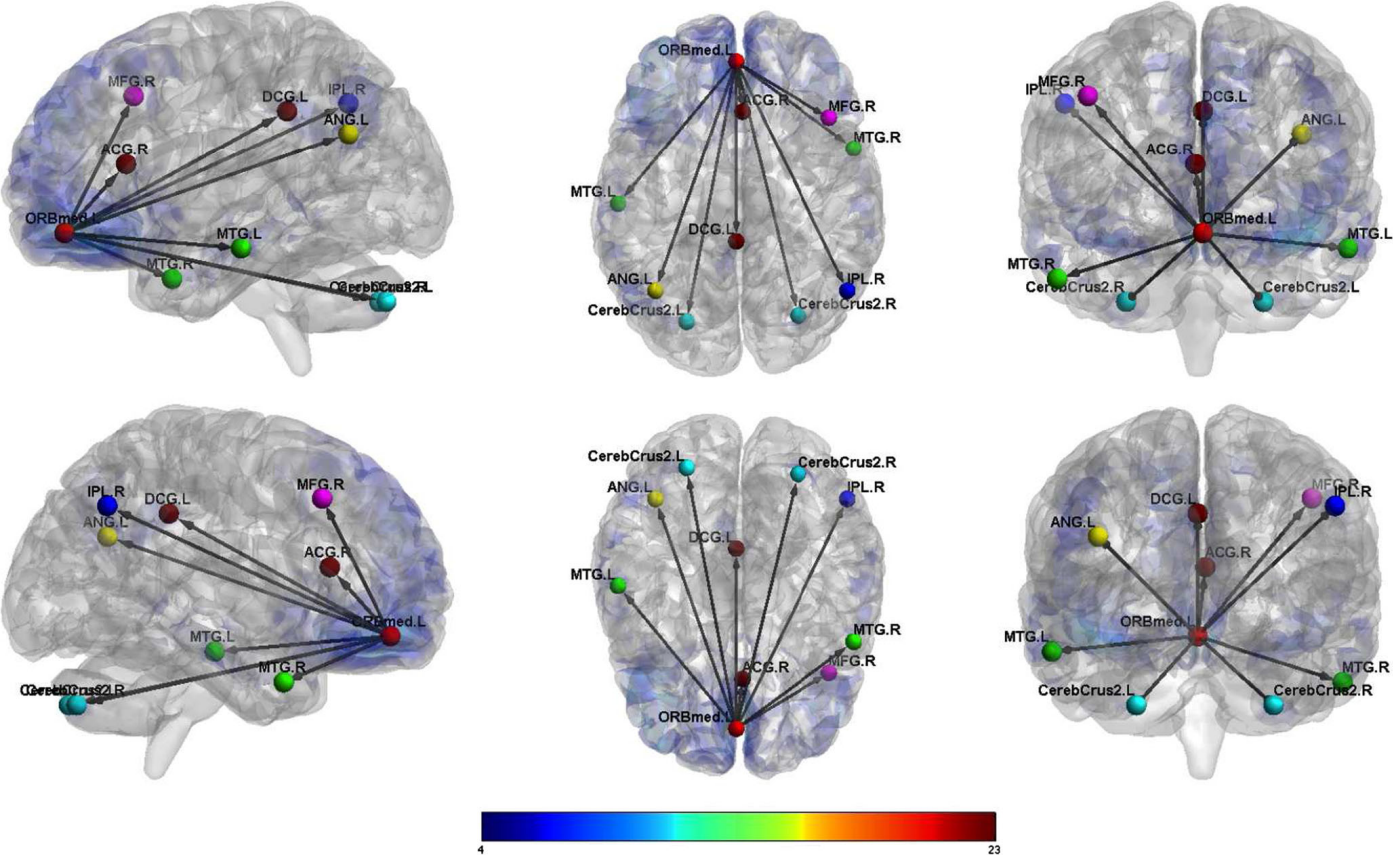
Male veterans with TBI: functional connectivity between left orbitofrontal cortex and other regions

Female veterans showed increased connectivity between the left OFC and the right cerebellum and between the left OFC and the right superior parietal cortex compared to male veterans. (See Table 4.) Female veterans also demonstrated increased connectivity, relative to males, between the right OFC and the right cerebellum and between the right OFC and right mid occipital cortex. (See Figs. 1 and 2.)

**Table 4:** Orbitofrontal connectivity for males with TBI vs females with TBI (*p*_FWEcorr_ < 0.05)

	Activated voxels (k)	Coordinates			
		x	y	z	T-score
Resting; females					
Left ORBF					
L Inf Orb Frontal	2139	−33	21	−12	14.06
L Mid Temporal	22	−63	−48	−6	7.21
L Inf Parietal	153	−48	−57	48	6.88
R Crus2 Cerebellum	65	33	−75	−45	6.14
L Sup Frontal	26	−21	30	60	6.13
L Precuneus	139	−9	−57	33	6.06
L Crus1 Cerebellum	54	−21	−87	−24	5.64
R Angular	37	54	−63	30	5.64
L Mid Temporal	22	−57	−21	−21	5.39
L Mid Temporal	31	−66	−30	−9	5.32
Medial Sup Frontal	22	9	54	18	5.11
Right ORBF					
R Inf Orb Frontal	2009	51	30	−12	12.46
L Mid Temporal	39	−66	−51	−9	7.73
L Crus2 Cerebellum	387	−18	−87	−48	7.37
L Inf Temporal	38	−48	−24	−18	7.37
L Angular	82	−39	−69	51	6.36
R Angular	95	45	−63	42	6.25
R Mid Frontal	28	42	24	54	5.80
R Inf Orb Frontal	21	21	12	−24	4.81
Resting; males					
Left ORBF					
L Med Orb Frontal	6103	0	48	−12	22.64
L Mid Temporal	566	−57	−21	−18	9.12
L Angular	418	−39	−63	27	8.78
R Mid Temporal	301	57	6	−30	8.46
R Inf Parietal	95	54	−63	39	7.66
L Mid Cingulum	193	0	−39	36	6.98
L Crus2 Cerebellum	70	−24	−78	−39	6.38
R Crus2 Cerebellum	124	30	−75	−39	6.03
R Ant Cingulum	21	3	24	15	4.82
R Mid Frontal	23	45	21	42	4.47
Right ORBF					
R Med Orb Frontal	6333	3	48	−15	16.64
R Mid Temporal	471	66	−21	−18	11.60
L Angular	270	−45	−72	39	9.06
R Inf Parietal	358	48	−57	51	8.36
L Mid Temporal	267	−63	−27	−15	8.06
L Crus2 Cerebellum	133	−27	−78	−39	6.06
L Inf Temporal	22	−57	−9	−33	5.99
R Mid Cingulum	132	12	−51	33	5.86
L Crus2 Cerebellum	45	−9	−81	−30	5.30
Resting; females > males					
Left ORBF					
R 8 Cerebellum	1081	6	−66	−30	4.19
R Sup Parietal	565	18	−51	57	3.97
Right ORBF					
R 6 Cerebellum	1266	30	−66	−21	4.54
R Mid Occipital	505	39	−72	21	3.95
Resting; males > females					
Left ORBF					
R Mid Frontal	1904	36	27	36	4.30
L Mid Temporal	659	−60	−36	−12	4.20
Right ORBF					
R Mid Frontal	618	33	27	18	4.62
L Insula	1269	−36	9	−12	4.59
R Ant Cingulum	547	3	21	18	4.19

**Fig. 3:**
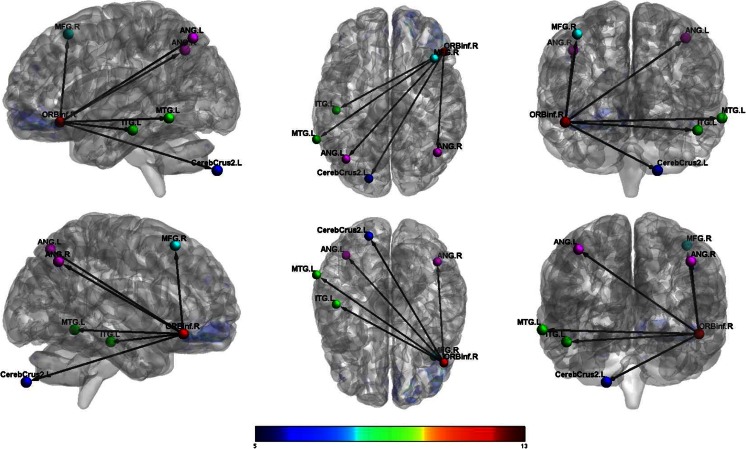
Female veterans with TBI: functional connectivity between right orbitofrontal cortex and other regions

**Fig. 4:**
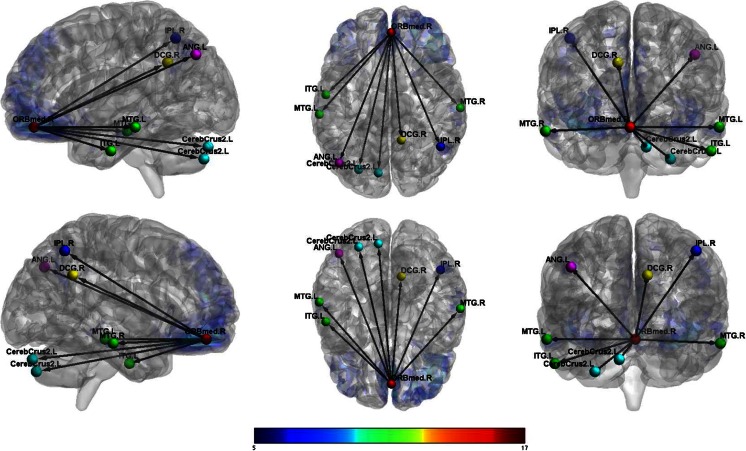
Male veterans with TBI: functional connectivity between right orbitofrontal cortex and other regions

Conversely, male veterans with TBI showed increased connectivity between the left OFC and the right mid frontal cortex and between the left OFC and the left mid temporal cortex compared to female veterans with TBI. Male veterans also showed increased connectivity between the right OFC and the right mid frontal cortex, the right OFC and the left insula, and the right OFC and the right anterior cingulum compared to female veterans. (See Figs. 3 and 4.)

### Regressions for OFC seeds with clinical measures

All significant regressions between OFC connectivity and clinical measures were only found in male veterans with TBI. A significant negative association was found for the overall score on the BPAQ Physical Aggression subscale and connectivity between the left OFC and the left angular region in male veterans. Increased physical aggression was associated with decreased connectivity between these regions in male veterans with TBI. There was also a positive relationship between the right OFC and the right cerebellum and right angular gyrus for the DAQ Revenge Planning scale in male veterans with TBI, reflecting higher revenge planning associated with increased connectivity between these. Additionally, connectivity between the right OFC and the right mid occipital cortex was negatively correlated with the DAQ Revenge Planning scale. None of these associations were significant for female veterans with TBI.

## Discussion

Based on prior research, we hypothesized that female veterans with TBI would differ from male veterans with TBI on OFC connectivity. This hypothesis was supported, as females showed greater connectivity between the left OFC and right cerebellum and right superior parietal regions and increased connectivity between the right OFC and right cerebellum and right mid occipital regions. In contrast, male veterans showed increased connectivity between the left OFC and right mid frontal and left mid temporal regions and increased connectivity between the right OFC and right mid frontal, left insula, and right anterior cingulum. Furthermore, we hypothesized that male and female veterans with TBI would differ in the strength of the associations between measures of aggression and functional connectivity originating from the OFC. This hypothesis also was supported, as only males showed significant correlations between measures of aggression and the functional networks studied. Specifically, these male participants showed decreased connectivity between the left OFC and left angular gyrus associated with increased physical aggression scores. Male veterans with TBI also demonstrated increased connectivity between the right OFC and the right cerebellum and right angular gyrus, which was associated with increased scores on revenge planning.

Male and female veterans with TBI also differed on aggression scores, as male veterans reported significantly more physical aggression and revenge planning compared to female veterans. While prior research on sex differences in temperament has been mixed with some studies presenting males as more aggressive (Maccoby and Jacklin 1974; Miles et al. 2015), the current findings expand upon this body of literature to show that well–characterized male veterans diagnosed with TBI using the OSU-TBI-ID, a standardized clinical assessment, reported more physical aggression based on responses to the BPAQ, which included a tendency or urge to engage in physical altercations, hit others, or participate in other types of physical violence. Additionally, compared to female veterans with TBI, male veterans trended toward higher scores on the DAQ measure of revenge planning, which included items such as holding a grudge and planning ways to retaliate against someone they felt had mistreated them. Based on these data, it appears there are sex-related differences in self-reported physical aggression and revenge planning.

The study groups appear similar in overall demographic and clinical presentation with no between group differences in age, education, race, number of children, or rank. Interestingly, female and male veterans differed on current marital status with more females being divorced and more males being married at time of assessment. However, when relationship status was collapsed to reflect whether individuals had ever been married, no between group differences were evident. Given that this is a cross-sectional study, it is difficult to determine the reasons for the difference in current relationship status. While scores on several of the clinical and mood measures did not differ at a statistically significant level, males reported 33 % higher depressive symptoms on the HAM-D and approximately 50 % more depressive symptoms on the POMS compared to the female veterans. Additionally, males reported having approximately 25 % more negative mood symptoms overall, as measured by the POMS total score. Given the sample sizes in this study, it remains to be seen whether mood differences will be statistically significant with the inclusion of more female and male veterans in future studies.

Imaging data from the current investigation utilized an atomically based seed region approach to the analysis of OFC connectivity in veterans with TBI. Recent studies applying resting state functional connectivity in individuals with TBI have found decreased functional connectivity in the default mode network (DMN) (Arenivas et al. 2014; Mayer et al. 2011). Some studies have identified an increase in functional connectivity in frontal portions of the DMN (Palacios et al. 2013) and reductions in functional connectivity in posterior portions of the DMN (Johnson et al. 2012; Zhou et al. 2012). Alterations in the connectivity between the DMN and other networks, including the task-positive network and the salience network also have been reported (Sours et al. 2013). However, findings from the current study are not directly comparable due to discrepancies in the methodology applied. For example, Mayer and colleagues (2011) used the rostral anterior cingulate cortex and posterior cingulate cortex to seed regions for an analysis of the default network, whereas the current investigation used left and right OFC seed regions and did not constrain the connections within a network.

Findings from this preliminary study indicated that male veterans with TBI demonstrated the strongest association between OFC connectivity and measures of aggression, suggesting these circuits may mediate behavioral response such as aggression. These findings are consistent with previous imaging studies that found a relationship between aggressive behavior and OFC metabolism in individuals with a history of physical aggression, and OFC blood flow changes in healthy individuals participating in an aggressive viewing task (New et al. 2007; Pietrini et al. 2000). As noted previously, the OFC has been shown to play a critical role in aggression (Antonucci et al. 2006; Beyer et al. 2014; Mehta and Beer 2010; Grafman et al. 1996; Miczek et al. 2007; Gansler et al. 2011), particularly when there are deficits in the top-down abilities of the OFC related to the inhibition of aggressive impulses (Siever 2008). Functional imaging studies have shown sex-related differences in OFC activation in response to a number of challenge tasks (Bolla et al. 2004; Goldstein et al. 2005). However, no study has reported sex-specific differences in OFC resting state connectivity.

One factor that may have contributed to the observed sex differences in connectivity and aggression is estrogen. Studies on the effects of estrogen therapy in women have shown effects on mood (Derman 1995; Joffe et al. 2006) while neuroimaging studies have reported differential brain activity during cognitive tasks, which were significantly associated with hormonal levels (Berman et al. 1997; Fernandez et al. 2003). Furthermore, a study of OFC activity in women during the presentation of emotional stimuli showed that estradiol levels were associated with brain activity levels (Protopopescu et al. 2005). Thus, it is possible that the sex- differences in connectivity observed in this study are associated with sex-specific brain patterns of structure and function that were present prior to concussive events. However, it may be that female brains are differentially impacted by TBI as recent investigations have suggested that there is a neuroprotective component to estrogen (Bramlett and Dietrich 2001; Day et al. 2013). Specifically, 17b-estradiol has been identified in several studies as positively influencing the outcome of both neurological and biochemical measures post-injury. The mechanism of action for how estrogen may act as a neuroprotective agent following TBI has not yet been fully characterized, although it has been hypothesized that estrogen acts on target cells including G-protein-linked estrogen receptor 1 (GPER) and one recent study reported acute protective effects of GPER post-TBI (Arevalo et al. 2015). These intriguing findings from animal studies raise important questions regarding sex differences in outcomes from TBI as well as potential avenues for new treatments; however, additional studies are needed to examine the role of sex differences in brain integrity and function.

Results from this preliminary study have important clinical implications, as they suggest that both female and male veterans with TBI would benefit from assessment for aggressive behavior. Current treatment recommendations for veterans with TBI and comorbid psychiatric symptoms include pharmacotherapy, cognitive behavioral therapy (CBT), cognitive processing therapy (CPT), behavior management techniques, stress inoculation training, and prolonged exposure (specifically in instances of PTSD) (Tanev et al. 2014; Wood and Thomas 2013). The study findings, however, identify female/male differences that may be important to consider in the treatment of individuals who have sustained TBI. Increased physical aggression and revenge planning could be a treatment focus for males more than females. Some males may need additional skills to cope with feelings that might trigger or break down defense mechanisms. Additionally, OFC function is related to a variety of behaviors beyond aggression, suggesting that some male veterans may need treatment focused on other OFC-related behaviors such as impulsivity. Sex differences in TBI and response to clinical interventions may be further examined using a longitudinal design wherein OFC connectivity and aggression are measured before and after a therapeutic intervention in order to quantify OFC connectivity changes related to treatment.

### Strengths and limitations

The present study included a number of strengths and limitations that should be considered when interpreting the findings. TBI and aggression are areas of great importance in the health and treatment of both female and male veterans, and knowledge that contributes to improved treatment and quality of care is imperative.

An important strength of this study is that comprehensive psychosocial interviews and assessment batteries were conducted in person by trained clinicians, and diagnostic assessments were derived via consensus. While the sample size is modest with 17 female and 24 male veterans participating in the investigation, similar rates of comorbid MDD and PTSD were found in both veteran TBI groups. The completion of the OSU-TBI-ID enabled use of standardized TBI definitions, including measures of severity of TBI and frequency of TBI. Further, while these data are preliminary in nature, they are based on a broad sample that includes both male and female veterans with a variety of comorbid health conditions allowing us to comment on behaviors affecting a general veteran population. However, there are also several important limitations of this study to consider. A significant portion of the clinical assessment was based on self-report and it is possible that either males or females may be more comfortable endorsing items via self-report. However, imaging outcomes were not self-report and would not be subject to similar biases, should they exist. Future studies will benefit from incorporating additional types of data including physiologic data, observational data, and experimental paradigms. Given the comorbidity of TBI, psychiatric diagnoses, and trauma, the heterogeneity of the population should be kept in mind, although a focus on aggressive traits rather than psychiatric diagnoses attempted to address this issue. Future studies will also benefit from inclusion of nonclinical groups to further elucidate not only sex differences but also differences between participants with TBI and comorbid psychiatric disorders and healthy control veterans.

In summary, the current study focused on sex differences in veterans with TBI, including differences in aggression and OFC connectivity. Male veterans with TBI reported increased physical aggression and trended toward increased revenge planning compared to female veterans with TBI. While females showed increased connectivity between the left and right OFC and parietal and occipital regions, males showed increased connectivity between the OFC and frontal and temporal regions. Moreover, resting state connectivity analyses showed significant relationships between the OFC and measures of aggression in males but not females. These data suggest that the neuropathological trajectory of TBI may differ in female and male veterans and be related to different clinical profiles, especially in aggression.
